# Collagen type I and decorin expression in tenocytes depend on the cell isolation method

**DOI:** 10.1186/1471-2474-13-140

**Published:** 2012-08-08

**Authors:** Markus U Wagenhäuser, Matthias F Pietschmann, Birte Sievers, Denitsa Docheva, Matthias Schieker, Volkmar Jansson, Peter E Müller

**Affiliations:** 1 Department of Orthopaedic Surgery, Ludwig-Maximilians-University Munich - Campus Grosshadern, Marchioninistr 15, 81377, Munich, Germany; 2 Experimental Surgery and Regenerative Medicine, Department of Surgery, Ludwig-Maximilians-University – Campus Innenstadt, Nußbaumstr 20, 80336, Munich, Germany

**Keywords:** Tenocytes, Tissue engineering, Isolation method, Gene expression

## Abstract

**Backround:**

The treatment of rotator cuff tears is still challenging. Tendon tissue engineering (TTE) might be an alternative in future. Tenocytes seem to be the most suitable cell type as they are easy to obtain and no differentiation *in vitro* is necessary. The aim of this study was to examine, if the long head of the biceps tendon (LHB) can deliver viable tenocytes for TTE. In this context, different isolation methods, such as enzymatic digestion (ED) and cell migration (CM), are investigated on differences in gene expression and cell morphology.

**Methods:**

Samples of the LHB were obtained from patients, who underwent surgery for primary shoulder arthroplasty. Using ED as isolation method, 0.2% collagenase I solution was used. Using CM as isolation method, small pieces of minced tendon were put into petri-dishes. After cell cultivation, RT-PCR was performed for collagen type I, collagen type III, decorin, tenascin-C, fibronectin, Scleraxis, tenomodulin, osteopontin and agreccan.

**Results:**

The total number of isolated cells, in relation to 1 g of native tissue, was 14 times higher using ED. The time interval for cell isolation was about 17 hours using ED and approximately 50 days using CM. Cell morphology *in vitro* was similar for both isolation techniques. Higher expression of collagen type I could be observed in tenocyte-like cell cultures (TLCC) using ED as isolation method (p < 0.05), however decorin expression was higher in TLCC using CM as isolation method (p < 0.05). Dedifferentiation potential seemed to be similar for both isolation techniques.

**Conclusion:**

In summary tenocyte-like cells can be obtained with both isolation methods (ED and CM) from the LHB. As no obvious disadvantage could be seen using ED, this method is more suitable for clinical use, as time for cell isolation is shorter and a remarkably higher number of cells can be obtained. However, both isolation methods can further be improved.

## Backround

Tendon and ligament injuries are common injuries in Orthopedics. The most important injuries are ruptures of the anterior cruciate ligament in the knee, rotator cuff tears in the shoulder and ruptures of the Achilles tendon [1].

Up to date, the treatment of severe rotator cuff tears remains challenging. The dimension of these ruptures often does not allow a complete reconstruction. Only specific groups of patients benefit from tendon transfer procedures. In Europe, allogenic tendons grafts are not used routinely [2]. Moreover, in 2006 Moore et al. advised not to use allogenic tendon grafts for rotator cuff reconstruction, because the clinical outcome was comparable to simple debridement and had an increased risk of infection and host-versus-graft-reaction [3].

In future, an alternative approach for irreparable rotator cuff tears might be tendon tissue engineering (TTE). It tries to create tendon tissue of good quality *in vitro* aiming to take over its specific function *in situ* after implantation. An important issue for the TTE is to find out which cell type is the most effective for *in vitro* cell seeding. The ideal cell type should fulfill certain criteria. Firstly, it should be possible to isolate cells from autologous tissue to avoid host-versus-graft reaction after implantation. Secondly, an adequate number of vital cells should be available after the cell isolation. Thirdly, these cells should have the ability to expand *in vitro* as well as to maintain phenotypic stability throughout the passages [4].

So far, fibroblasts, mesenchymal stem cells and tenocytes have been investigated [5-7]. Tenocytes might be the most suitable cells as no differentiation during *in vitro* cultivation is necessary. Tenocytes have been shown to grow *in vitro* without signs of senescence [8]. There is also evidence that cell proliferation of tenocytes *in vitro* is comparable to mesenchymal stem cells [9].

In this context, the long head of the biceps tendon (LHB) seems to be promising for tenocyte isolation. A (partial or complete) rupture of the LHB is often found in association with rotator cuff tears [10,11]. For this reason tenotomy of the LHB is performed regularly during rotator cuff reconstruction [12-14]. Donor side morbidity after tenotomy of the LHB is negligible and patient satisfaction is high [15].

There are two different methods to isolate tenocytes from tendon tissue. Tenocytes can be isolated using enzymatic digestion (ED) of the extracellular matrix compounds [6,16]. Another alternative is to obtained tenocytes by cell migration (CM) as cells migrate out of the tissue explants after culturing in cell culture medium [17].

Tenocytes synthesize specific proteins of the extracellular matrix, which has a highly ordered composition [18]. Collagen type I, collagen type III, decorin, tenascin-C are fundamental proteins in the extracellular matrix of tendons. High expression levels of collagen type I and decorin seem to be essential for cells to be suitable for TTE applications, as they play an important role in tissue formation [19,20]. Scleraxis and tenomodulin are commonly used as markers for tenogenic differentiation [21-23].

Tenascin-C and fibronectin are two glycoproteins of the extracellular matrix, which are essential for cell-cell and cell-matrix interactions [24]. Aggrecan and osteopontin are located predominantly in the extracellular matrix of cartilage and bone.

The aim of this study was to examine if the LHB is suitable as cell source for TTE. Additionally, we compared two different isolation methods, ED and CM. In order to investigate the influence of these isolation methods on tenocyte-like cell cultures (TLCC), we analyzed cell morphology and gene expression. We hypothesized that both isolation methods deliver similar cell yield and show no differences in gene expression.

## Methods

### Cell isolation procedure

Tendon samples were obtained from patients, who underwent surgery for a primary shoulder arthroplasty. Surgery was performed by the senior author (P.E.M), an experienced shoulder surgeon. The study was carried out following the regulations of the Medical Center Ethics Committee of the Ludwig-Maximilians-University of Munich (ethical grant number: 063–09).

Altogether, seven tissue samples of the LHB were collected from patients with an average age of 60 years (± 8.9 years). Tendon samples were carried to the laboratory in cell culture medium (DMEM/HAM’s F12, Biochrom, Berlin, Germany) where cell isolation was immediately performed. Under sterile conditions tendons were washed three times with PBS buffer (Biochrom, Berlin, Germany) and cut into small pieces. For the following cell isolation previously published methods were used [6,16,17]. Briefly, the sheath and surrounding paratenon were removed and the tendons were minced into small pieces. At this stage, slices from each sample were randomly assigned to perform the following isolation procedure.

#### ED method

One part of the tendon slices was incubated in 0.2% collagenase I solution (Sigma-Aldrich, Steinheim, Germany) for approximately 18 hours in 37°C, CO_2_ 5%. After digestion, cells were filtered (100 μm) (BD Biosciences, Erembodegem, Belgium), the suspension was washed three times with PBS buffer and centrifuged (Heraeus, Hanau, Germany) at 300 g for 5 minutes. Before cell cultivation, tenocytes were counted using a heamocytometer and trypan blue staining to distinguish between dead and vital cells.

#### CM method

The other part of the tendon slices were placed into petri-dishes filled with 10 ml cell culture medium (DMEM/HAM’s F12), supplemented with 10% FCS, and were subcultivated (37°C, CO_2_ 5%). Culture medium was changed every second day. After a few days, the first colonies of migrating tenocytes around the slices could be seen. After approximately 3 weeks, the tendon slices were carefully transferred into new petri-dishes. Altogether three migration cycles were performed. Before cell cultivation the total number of cells was counted using a haemocytometer and trypan blue.

### Tendon cell cultures

The isolated tenocytes were placed in culture flask in DMEM/HAM`s F12 (1:1) supplemented with 10% FCS, 2 mM L-Glutamin, ascorbic acid 1250 μg/ml, amino acids, Penicillin/Streptomycin 60 μg/ml and Amphotericin B 25 ng/ml (Biochrom, Berlin, Germany). The seeding density for each isolation method was as follows: CM-714 cells/cm^2^ and ED-2857 cells/cm^2^. As soon as the cultured cells reached 80-90% confluence, they were treated with 0.05% trypsin/0.02% ethylenediaminetetraaciticacid (EDTA) (Biochrom, Berlin, Germany) and subcultured. Osteoblasts (HOB lot: 540X130406) and fibroblasts (HFIB lot: 055 H170100) (Provitro, Berlin, Germany) were subcultured in DMEM cell culture medium. Supplements were the same as for TLCCs but without Amphotericin B. Chondrocytes were obtained from patients, who underwent total knee replacement, as previously descibed by us [25]. Morphological cell assessment was performed using a phase-contrast microscope (Zeiss, Munich, Germany). Total cell number was calculated after every cell passage.

### rt-pcr

Tenocytes in the third passage were homogenized with lysis buffer (Quiagen, Hilden, Germany) using a QIAshredder (Quiagen) and a centrifuge at 8000 g for 3 minutes. For further purification of RNA, the RNeasy Mini Kit (Quiagen) was used following the manufacturer’s manual. Leftovers of DNA were digested on-column with RNAse-free DNase I Set (Quiagen) for 15 minutes. cDNA was synthesized by using Reverse Transcription System Set (Promega, Mannheim, Germany) following the manufacturer`s instruction. Briefly, 1 μg of RNA, 4 μl of MgCl_2,_ 2 μl of 10x Reactionbuffer, 2 μl 10 mM Deoxynucleotidetriphosphate (dNTP)-mix, 0.5 μl of RNase-inhibitor, 0.6 μl Reverse Transcriptase, 0.5 μl Randomprimer and RNase-free water were mixed up to a final volume of 20 μl. Samples were incubated for 10 minutes at 25°C, following incubation at 42°C for 60 minutes. cDNA samples were stored at −20°C until further use.

RT-PCR was performed for collagen type I, decorin, collagen type III, tenascin-C, fibronectin, Scleraxis, tenomodulin, osteopontin and aggrecan. Sequences and product lengths are shown in Table 1[17,26,27]. For the RT-PCR, 1 μl of cDNA was mixed with 0,5 μl of forward and reverse primers (each 0.5 μM), 10x PCR buffer (100 mM Tris–HCl, pH 8.8 at 25°C, 500 mM KCl, 0.8% [v/v] Nonidet P40), 0,2 μl of Deoxynucleotidetriphosphate (dNTP)-mix (each 2 mM), 1 μl MgCl_2_ (25 mM), 0,1 μl of taq-polymerase (5 u/μl) (Fermentas, St. Leon-Rot, Germany) and filled up to a final volume of 20 μl with RNase free water. The reaction mixtures were incubated for 3 min at 95°C, followed by 30 sec at 95°C, 45 sec at 50-64° and 1 min at 72°C up to 35 cycles, and then 10 min at 72°C. Products were analyzed by 2% gel-electrophoresis. To control results, at least two independent experiments were performed for all seven samples.

**Table 1:** RT-PCR primer sequences and product length

**Target gene**	**Primer sequence**	**Length**
1. GAPDH [NM**_**002046]	Sense: GAGTCCACTGGCGTCTCCAC	188 bp
	Antisense: GGTGCTAAGCAGTTGGTGGT	
2. collagen Typ I, alpha 1 [NM_000088]	Sense: GGCCCAGAAGAACTGGTACA	200 bp
	Antisense: GGCTGTTCTTGCAGTGGTAG	
3. collagen Typ III, alpha 1 [NM_033150]	Sense: CCAGGAGCTAACGGTCTCAG	103 bp
	Antisense. CAGGGTTTCCATCTCTTCCA	
4. decorin [NM_001920]	Sense: TGCTGTTGACAATGGCTCTC	192 bp
	Antisense: GCCTTTTTGGTGTTGTGTCC	
5. fibronectin [NM_212475]	Sense: ATGATGAGGTGCACGTGTGT	135 bp
	Antisense: CTCTTCATGACGCTTGTGGA	
6. tenascin-C [NM_002160]	Sense: TCAAGGCTGCTACGCCTTAT	230 bp
	Antisense: GTTCTGGGCTGCCTCTACTG	
7. Scleraxis [17]	Sense: CCTGAACATCTGGGAAATTTTAC	111 bp
	Antisense: CGCCAAGGCACCTCCTT	
8. tenomodulin [NM_022144]	Sense: CCATGCTGGATGAGAGAGGT	123 bp
	Antisense: CTCGTCCTCCTTGGTAGCAG	
9. osteopontin [26]	Sense: TTGCTTTTGCCTCCTAGGCA	430 bp
	Antisense: GTGAAAACTTCGGTTGCTGG	
10. aggrecan [27]	Sense: CACTGTTACCGCCACTTCCC	183 bp
	Antisense: ACCAGCGGAAGTCCCCTTCG	

### qrt-pcr

The gene expression of collagen type I alpha I and decorin was also determined by quantitative RT-PCR (qRT-PCR) using a light cycler instrument 2.0 (Roche Diagnostic, Mannheim, Germany). Target sequences were amplified using LightCycler Primer Sets (Search LC, Heidelberg, Germany) with LightCycler-Fast Start DNA Master SYBR Green I Mix (Roche Applied Science, Mannheim, Germany) following the manufacture`s manual. Reactions were performed in doublets. At least two independent experiments were performed for all seven samples and difference in efficiency had to be less than 0.05. For relative quantification of the gene expression, samples were normalized to cyclophilin B. Primer sequences are property of Search LC, Heidelberg, Germany and cannot be mentioned in this paper. (Lot numbers: Cyclophilin B: 120906, 090408, collagen type I alpha 1: 290606, 020608, dcorin: 140508).

### Statistics

Data is shown as mean ± SD. Statistical analysis was performed using GraphPad Prism 3.0 (San Diego CA, USA). The Mann–Whitney test was used to analyze significant differences between the groups (ED and CM). For comparison of multiple time points in one group, the Friedman test for multivariate analysis and Dunn’s multiple comparison tests were used. Both tests are designed for paired samples. Level of significance was set up p < 0.05.

## Results

### Cell yield was significantly higher with enzymatic digestion (ED) method

**Figure 1:**
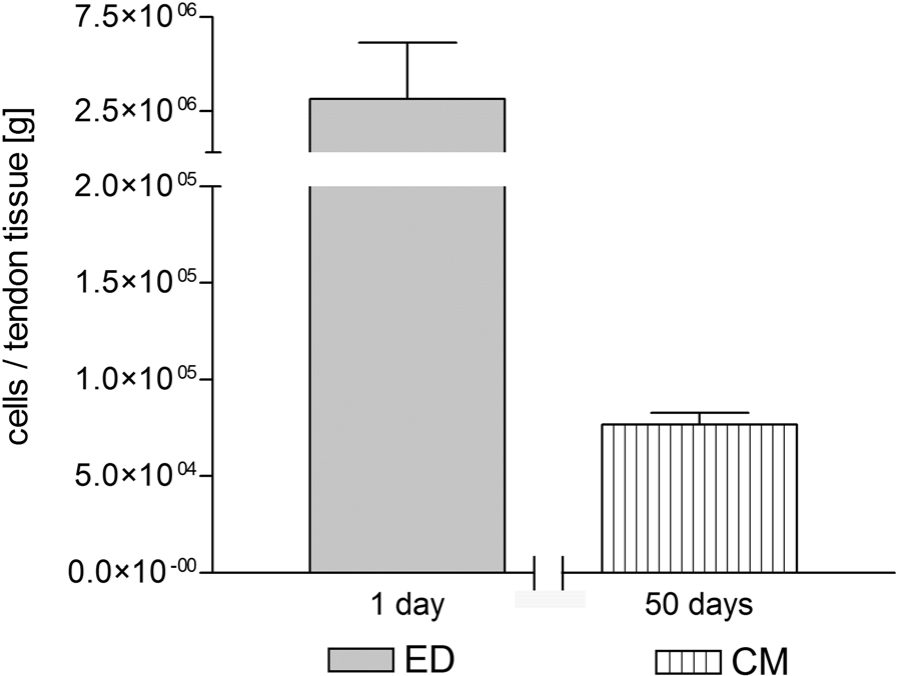
**Yield of cells.** Cell yield after both isolation methods and normalized to 1 g of native tendon tissue. Cells were counted using a heamocytometer and trypan blue. The amount of cells after 1 day using ED is 14 times higher than using CM after 50 days.

For cell isolation using CM, 0.11 g ± 0.09 g of the LHB was used. Respectively, for isolation using ED 0.337 g ± 0.19 g of tendon tissue was used. As we expected a higher loss of cells due to enzymatic treatment we used three times more tissue for ED compared to CM. After seven weeks, an average of 75x10^3^ cells could be generated using CM. Using ED as isolation method an average of 3.15x10^6^ cells could be isolated with approximately 17 hours. In relation to 1 g of the LHB, CM could generate 6.70x10^5^ cells while ED could generate 9.35x10^6^ cells, meaning a ratio 1:14 (CM:ED). An overview over time and cell yield gives Figure 1.

### Cell morphology was similar for both isolation methods

**Figure 2:**
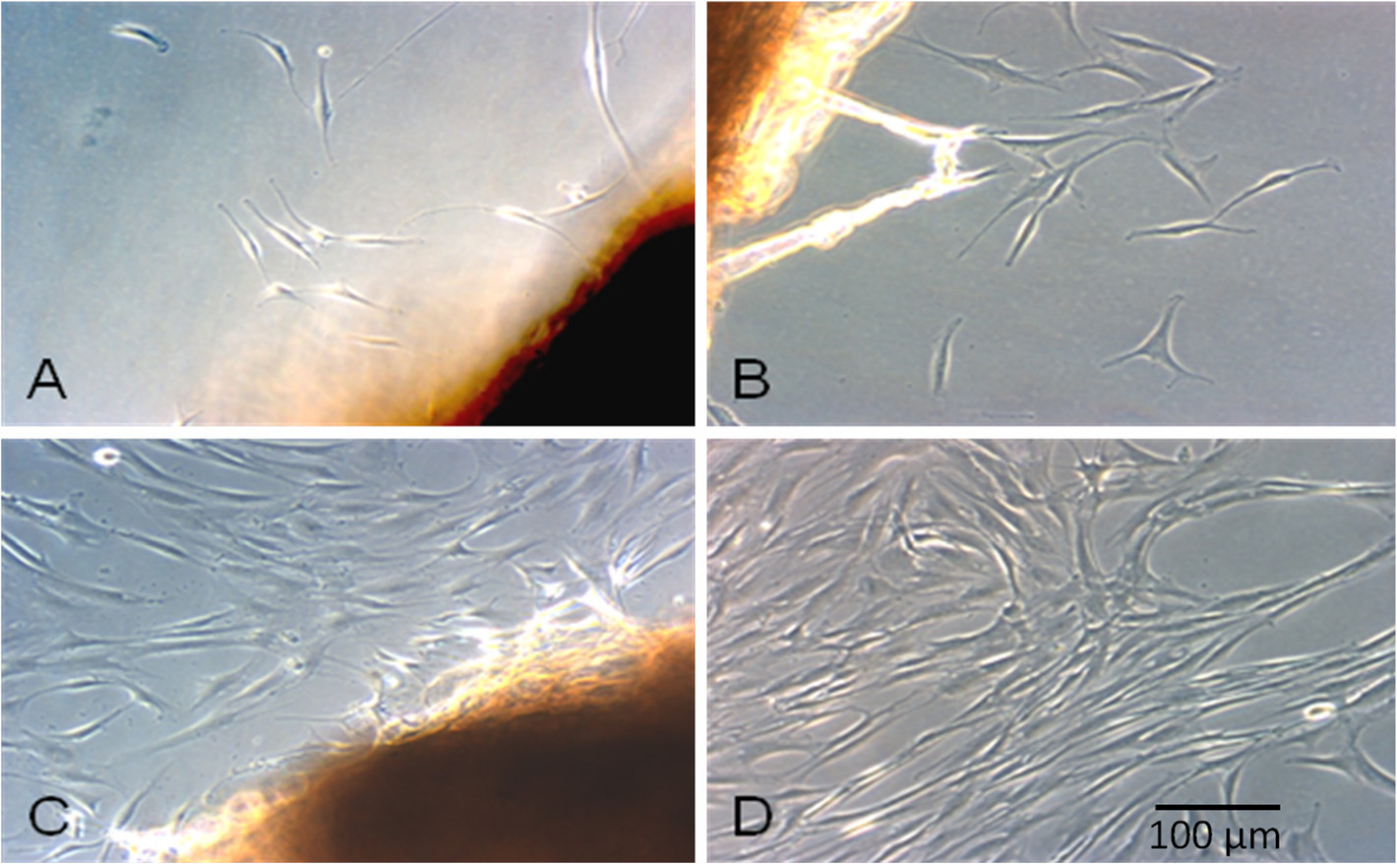
**Migrating tenocytes of the LHB.** The tendon slices were put into petri-dishes, incubated (37°C, 5% CO2) in cell culture medium (DMEM/HAM’s F12) and analyzed by light microscopy. An increasing amount of spindle-like cells could be observed at different time points. **A** = after 8 days, **B** = after 11 days, **C** = after 16 days **D** = after 22 days.

First migrating cells could be seen around the tendon slices after one week. With increasing culture time these colonies became denser and reached a confluence of about 90% after three weeks. Accelerated cell migration could be observed during the following cell migration cycles (Figure 2).

**Figure 3:**
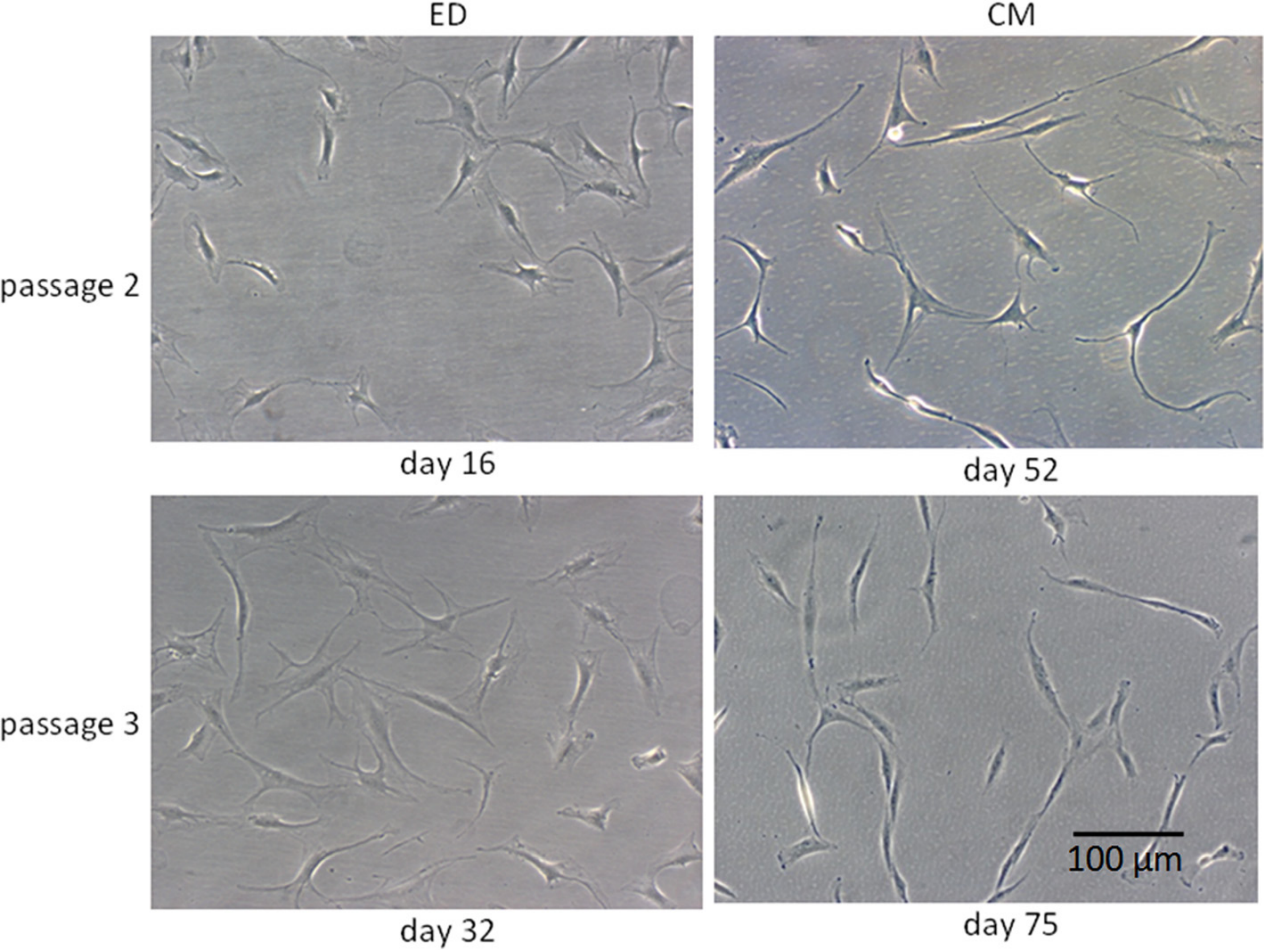
**Cell morphology in TLCC.** Tenocyte cell cultures at different time of subcultering. ED (enzymatic digestion) CM (cell migration). Cells of the second and third cell passage are shown. Cells show typical morpholpgy for tenocytes and no differences between both methods.

**Figure 4:**
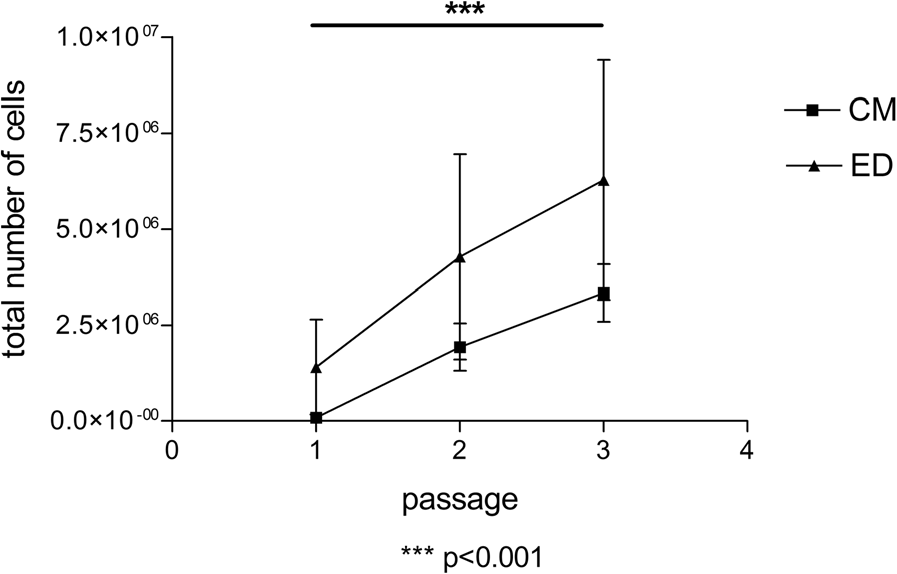
**Cell proliferation.** Absolute number of cells from passage 1 to 3 (n = 7). Proliferation was almost similar for TLCC of both isolation methods, however yield of cell and proliferation rate seemed to be more variable for ED. Cell number significantly increases from passage 1 to 3 for both groups, ED and CM.

Figure 3 shows TLCCs of the second and third passage. Cell morphology was similar for both groups investigated. Even in the fourth passage, cells exhibited elongated shapes and several membrane protrusions. Over time, however, the amount of polygonal-shaped cells increased in both groups. Overall, the number of cells significantly increased from passage 1 to 3 (p < 0.001) in both groups. However yield of cell and proliferation seemed to be more variable for ED. (Figure 4).

### Gene expression analysis showed comparable results in both groups

**Figure 5:**
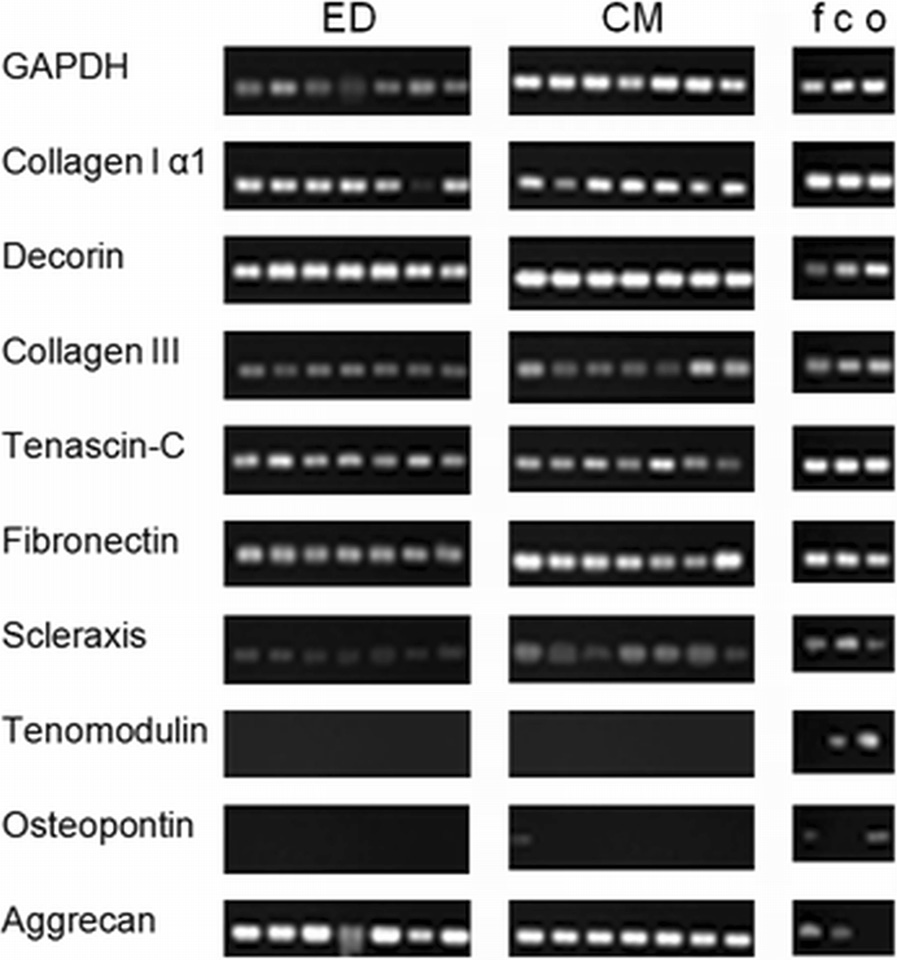
**RT-PCR analysis.** Cells in the third cell passage were used. cDNA from other musculoskeletal cell types such as f, fibroblasts (passage 6), c, chondrocytes (passage 3) and o, osteoblasts (passage 2) were used for comparison. GAPDH was used for normalization. ED, enzymatic digestion, CM, cell migration. Each band represents one patient/LHB (n = 7).

To explore differences between the two isolation methods, the expression of main compounds of the extracellular matrix and tenogenic differentiation markers (Scleraxis and tenomodulin) were analyzed using RT-PCR. Results are shown in Figure 5.

The expression of collagen type I alpha I, collagen type III and decorin could be detected in TLCCs of both isolation methods. Cells of other musculoskeletal tissues also showed positive results on these three genes (line 2–4).

The expression of tenascin-C and fibronectin could be seen in all TLCC (line 5–6). The expression of Scleraxis was detected in all TLCC, too (line 7). Interestingly fibroblasts, chondrocytes and osteoblasts also showed Scleraxis expression. The expression of tenomodulin, could not be detected in any TLCC. Here, tenomodulin expression could be detected in chondrocytes and osteoblasts (line 8). Osteopontin was not expressed in TLCC (line 9), but could be seen in osteoblast cultures. Interestingly, the expression of aggrecan, could be observed in all TLCCs. Its expression was also seen in chondrocytes (line 10).

### qRT-PCR of collagen type I alpha I and decorin showed different expression in both groups

**Figure 6:**
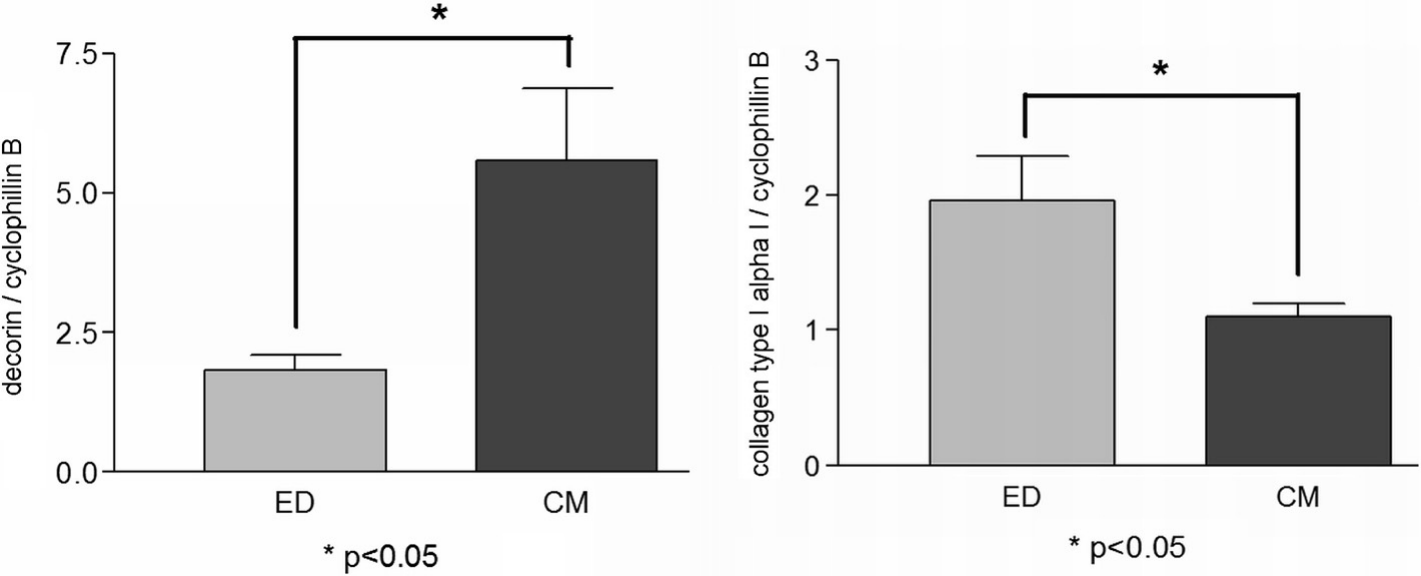
**Quantitative RT-PCR.** RT-PCR was performed for collagen type I and decorin. Relative gene expression was estimated against cyclophilin B.

Our results showed that the expression of collagen type I alpha I was higher in TLCC using ED as isolation method (≤0.05). In contrast, decorin showed higher expression in TLCC using CM as isolation method (≤0.05) (Figure 6). Collagen type I expression and decorin expression was about the same level in TLCC isolated by ED. If TLCCs were gained using CM, decorin expression was about five times higher than collagen type I expression.

## Discussion

It is important that TTE procedures, which aim to replace damaged tissue, only cause as little donor side morbidity as possible. The use of the LHB in TTE might be promising. It is removed regularly during shoulder operation as it often causes inflammation and pain. The loss of function is still under debate but in most cases the removal of this tendon is well tolerated by the patients [12-15,28].

Two different isolation methods can be used in TTE (ED and CM). The aim of this study was to compare these two methods with regards to the quality of generated TLCCs. ED uses the enzyme collagenase I to separate cells from their extracellular matrix. The period of time to obtain cells is quite short (17 hours). Another advantage of this method is a higher cell yield compared to CM. This makes the method suitable for clinical use. CM is an alternative to isolate tenocytes from the LHB. A clear disadvantage of this method is that cell yield is poor and takes remarkably longer than using ED (1–2 weeks) [17]. In our study, a reasonable number of cells could be obtained only after three migration cycles which lasted 6–8 weeks altogether. Our results showed that in relation to 1 g of native tissue ED could generate 14 times more cells than CM. Since, cell yield and the isolation time are essential for clinical use, this isolation method will not be clinically appropriate in nearer future.

It has been shown that tendons contain cells different from tenocytes. These cells are named tendon stem and progenitor cells (TSPC) [29-31]. We assumed that ED leads to obtaining both, tenocytes and TSPC, whereas cell migration tends to favor mature tenocytes. Therefore, we think that this might be an explanation for higher total number of cells in TLCCs isolated using ED throughout the passages as well as for higher variability of cell proliferation.

Cell morphology seemed to be similar in TLCCs no matter which isolation method was used. TLCCs of both isolation methods showed a significant increase in total cell number during passaging, illustrating a comparable cell-viability and -function.

All important structural compounds of the extracellular matrix in tendon such as collagen type I, collagen type III, decorin, tenascin-C and fibronectin were expressed continuously by all TLCC of the third cell passage. No difference could be detected between the two different isolation methods. Importantly, the transcription factor Scleraxis was expressed by all TLCC. It has been shown to play an important role in coordinating the response to injury in the pathogenesis of tendon disorders [32]. In contrast, tenomodulin expression could not be detected in TLCCs demonstrating a loss of this marker during passaging. This is in line with other studies by Yao, L. et al. [33] and Jelinsky et al. [34], which also showed a loss of tenomodulin expression in two-dimensional culture systems.

The detection of aggrecan could be evidence for chondrogenic differentiation in TLCC [35]. In this study, we observed aggrecan expression in TLCC of both isolation methods. Former research could show that the use of collagenase type I has a negative effect on the differentiation of tenocytes. Lui, P. et al. could show, that the injection of collagenase I in the patellar tendon leads to ectopic ossifications and to a chondrogenic gene expression profile [36]. This might also explain the expression of aggrecan in TLCC in this study, as the same enzyme was used for ED. As this enzyme was not used for CM isolation method we assume that aggrecan expression in these cultures might give evidence for a chondrogenic drift during cell culturing. High expression of aggrecan has been shown in degenerative tendons, which we used as a cell source. This could also be an explanation for our findings [37,38]. However, investigating aggrecan expression in at least two separate cell passages is necessary for clarification. The osteogenic marker osteopontin could not be detected in any TLCC, no matter which isolation method was used. Osteogenic differentiation seemed unlikely.

Collagen type I and decorin are both essential proteins in the tendon tissue [19,20], therefore we analyzed their expression levels by quantitative PCR. Our results showed that collagen type I expression was higher in TLCCs using ED. An influence on collagen type I expression by external applications, such as laser irradiation and shock waves was shown by Chen, C. H. et al. and Bosch, G. et al. [39,40]. Therefore, we assume that the use of collagenase type I, another external application, during cell isolation might up-regulate collagen type I expression. An increased expression of decorin could be seen in TLCC using CM. Findings of Karousou, E. et al. and Corps, A. N. et al. [37,41] indicate that decorin is up-regulated after tendon rupture. These findings were confirmed by Yokota et al., as they showed that the decorin expression in ruptured supraspinatues tendons is upregulated, too. This effect even aggravates during cell cultivation [38]. During the isolation process an artificial tendon rupture was generated. This might explain the higher expression of decorin in TLCCs isolated using CM. Ratio of mature tenocytes and TSPC might differ using different isolation methods and could generally be a possible explanation for the differences in collagen I/decorin expression.

## Conclusion

In summary, it is possible to isolate cells from the LHB using both isolation methods (ED and CM). Since no obvious disadvantage could be seen in morphological cell assesment of cells isolated by ED, this method seems to be more suitable for clinical use. The fact, that the intervening period between tendon explantation and cell seeding is remarkably shorter using ED compared to CM, increasing its clinical suitability. In addition to that, the high cell yield and collagen type I expression is another advantage of the ED isolation method. However, both methods need further optimization.

## Abbreviations

CM: Cell migration; ED: Ezymatic digestion; LHB: Long head of the biceps tendon; TSPC: Tendon stem and progenitor cells; TLCC: Tenocyte-like cell culture; TTE: Tendon tissue engineering.

## Competing interests

The authors declare that they have no competing interests.

## Authors’ contributions

MW and MP carried out the molecular experiments and drafted the manuscript. BS and DD helped with the establishment of RT-PCR and participated in the design of the study. MS conceived of the study and helped with the study design. PM and VJ participated in the coordination and helped to draft the manuscript. All authors have read the manuscript.

## Pre-publication history

The pre-publication history for this paper can be accessed here:

http://www.biomedcentral.com/1471-2474/13/140/prepub
